# Concomitant medication use and clinical outcome of repetitive Transcranial Magnetic Stimulation (rTMS) treatment of Major Depressive Disorder

**DOI:** 10.1002/brb3.1275

**Published:** 2019-04-02

**Authors:** Aimee M. Hunter, Michael J. Minzenberg, Ian A. Cook, David E. Krantz, Jennifer G. Levitt, Natalie M. Rotstein, Shweta A. Chawla, Andrew F. Leuchter

**Affiliations:** ^1^ Department of Psychiatry and Biobehavioral Sciences, David Geffen School of Medicine at UCLA University of California Los Angeles Los Angeles California; ^2^ Laboratory of Brain, Behavior, and Pharmacology and the TMS Clinical and Research Program, Neuromodulation Division, Semel Institute for Neuroscience and Human Behavior University of California Los Angeles Los Angeles California; ^3^ Department of Bioengineering University of California Los Angeles Los Angeles California

**Keywords:** adrenergic, benzodiazepines, gamma‐aminobutyric acid (GABA), Major Depressive Disorder, psychostimulants, repetitive transcranial magnetic stimulation (rTMS), treatment outcome

## Abstract

**Background:**

Repetitive Transcranial Magnetic Stimulation (rTMS) is commonly administered to Major Depressive Disorder (MDD) patients taking psychotropic medications, yet the effects on treatment outcomes remain unknown. We explored how concomitant medication use relates to clinical response to a standard course of rTMS.

**Methods:**

Medications were tabulated for 181 MDD patients who underwent a six‐week rTMS treatment course. All patients received 10 Hz rTMS administered to left dorsolateral prefrontal cortex (DLPFC), with 1 Hz administered to right DLPFC in patients with inadequate response to and/or intolerance of left‐sided stimulation. Primary outcomes were change in Inventory of Depressive Symptomatology Self Report (IDS‐SR30) total score after 2, 4, and 6 weeks.

**Results:**

Use of benzodiazepines was associated with less improvement at week 2, whereas use of psychostimulants was associated with greater improvement at week 2 and across 6 weeks. These effects were significant controlling for baseline variables including age, overall symptom severity, and severity of anxiety symptoms. Response rates at week 6 were lower in benzodiazepine users versus non‐users (16.4% vs. 35.5%, *p* = 0.008), and higher in psychostimulant users versus non‐users (39.2% vs. 22.0%, *p* = 0.02).

**Conclusions:**

Concomitant medication use may impact rTMS treatment outcome. While the differences reported here could be considered clinically significant, results were not corrected for multiple comparisons and findings should be replicated before clinicians incorporate the evidence into clinical practice. Prospective, hypothesis‐based treatment studies will aid in determining causal relationships between medication treatments and outcome.

## 1 INTRODUCTION

Repetitive Transcranial Magnetic Stimulation (rTMS) has gained increasingly widespread clinical use for treatment of Major Depressive Disorder (MDD) since it was approved in the US in 2008 for treatment of antidepressant drug‐resistant unipolar major depression. Although initial trials examined the efficacy of rTMS monotherapy for MDD (George et al., 2010; Levkovitz et al., 2015; O'Reardon et al., 2007), in clinical practice physicians typically administer rTMS as an augmentation therapy, rather than discontinue a patient's current medications. Several studies have confirmed the clinical effectiveness of rTMS in patients who are taking adjunctive antidepressants (Carpenter et al., 2012; George, Taylor, & Short, 2013) or have examined the effects of initiating rTMS for a brief period at the outset of treatment with antidepressant medication as a means of bolstering medication response (Berlim, den Eynde Van, & Daskalakis, 2013). However, none have examined the effects of different classes of medication on rTMS treatment outcome.

Clinical guidelines presently address concomitant medication use solely from a safety perspective. Caution is advised when administering rTMS to patients who are taking stimulants or other medications that may lower seizure threshold, or following a decrease or discontinuation of antiepileptics, benzodiazepines, or other medications with anticonvulsant properties (McClintock et al., 2018). A recent consensus states that “TMS therapy can be administered in the presence or absence of concurrent antidepressant or other psychotropic medications” (Perera et al., 2016). Although there are no reports of rTMS clinical outcomes in relationship to concurrent medications, experimental pharmaco‐TMS‐EEG studies in single‐ or paired‐pulse paradigms have routinely demonstrated effects of central nervous system drugs on measures of cortical excitability, connectivity, and plasticity (Ziemann, 2004). Given that therapeutic effects of rTMS are posited to result from long‐term potentiation (LTP) or long‐term depression (LTD)‐like effects in critical brain circuits (Kobayashi et al., 2017; Ziemann et al., 2015), it is reasonable to hypothesize that concurrent psychotropic medications could influence clinical outcome.

The present study explored associations between concomitant medications and rTMS outcome during treatment of MDD. The motivation for this investigation was twofold: first, to examine evidence that could help inform clinical decision‐making when addressing the integration of psychopharmacology with rTMS; and second, to identify those medication mechanisms of action (MOAs) that might help elucidate which classes of psychotropic drugs are most likely to interact with rTMS effects. We therefore examined the effect of standard medication classes on treatment outcome in 181 patients receiving a standard clinical course of rTMS. In supplementary analyses (Data S1), we also examined the effects of medication on outcome using a novel MOA‐based schema based on the neurochemical actions of individual drugs. Results identified categories of medication use that were associated with greater or lesser clinical improvement over the course of rTMS.

## 2 METHOD

### 2.1 Overview and subjects

This retrospective chart study was undertaken to examine potential relationships between categories of medication use and clinical outcome to rTMS treatment for depression. There were no experimental manipulations; rTMS treatment and medication data collection were performed naturalistically.

**Figure 1:**
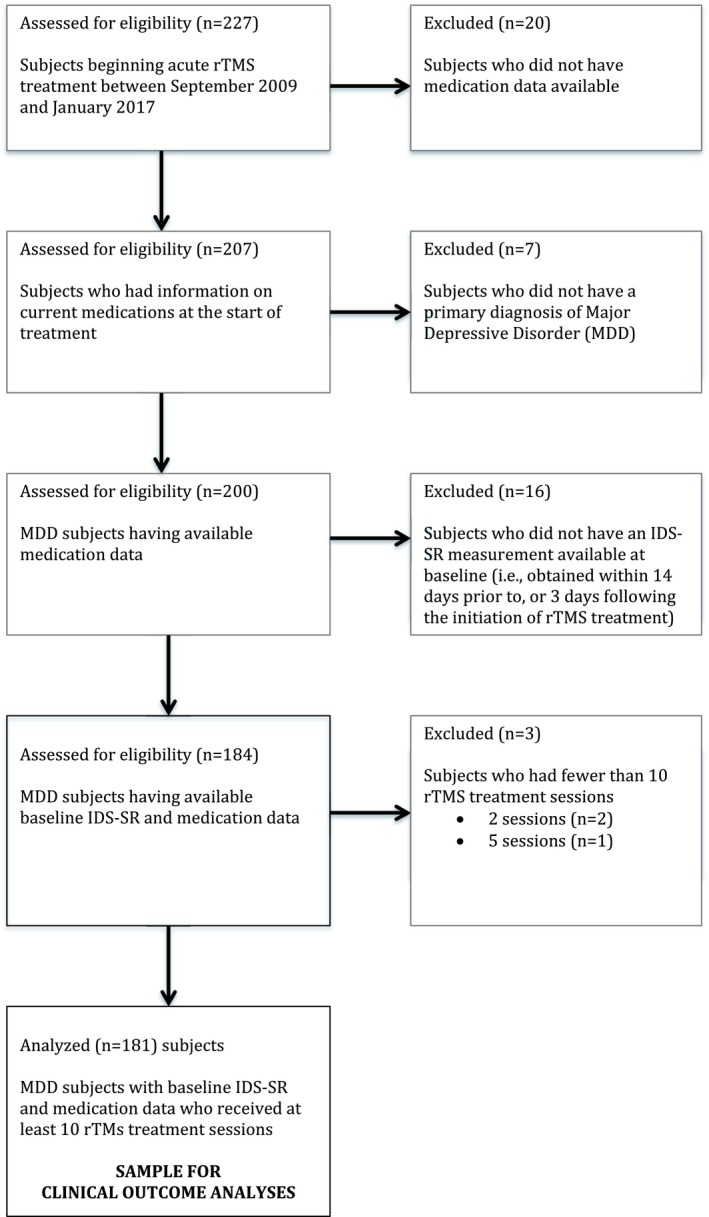
Data flow diagram of subject inclusion

Subjects (*n* = 227) were all patients treated in the TMS UCLA Clinical and Research Program between September 2009 and January 2017 and who provided written informed consent to participate in this UCLA IRB‐approved study. Subjects were treated in accordance with the 2013 Declaration of Helsinki. Analyses included subjects who had baseline medication data available, received at least 10 rTMS treatment sessions for non‐psychotic MDD, and were assessed at baseline using the 30‐item Inventory of Depressive Symptomatology Self Report (IDS‐SR30) (Rush, Gullion, Basco, Jarrett, & Trivedi, 1996). Figure 1 shows a data flow diagram of the analyzable sample (*n* = 181).

### 2.2 Medication categories

Medications were classified according to drug classes (Table 1) using 13 mutually exclusive categories: Selective Serotonin Reuptake Inhibitors (SSRI); Serotonin‐Norepinephrine Reuptake Inhibitors (SNRI); Tricyclic antidepressants (TCA); Monoamine oxidase inhibitors (MAOIs); Atypical antidepressants; Atypical antipsychotics; Typical antipsychotics; Antiepileptics (AED); Benzodiazepines (BDZ); Quasi‐benzodiazepines (QBDZ); Psychostimulants; Lithium; and “Other.” The “Other” medication class was heterogeneous and included opiates, thyroid, melatonin, and memantine. These medications do not have clear effects on cortical excitability or plasticity, and excepting thyroid hormone, they lack established antidepressant augmentation efficacy. Only seven subjects were taking thyroid hormone at study entry. An alternate classification schema also examined outcome in relation to use of medications grouped into eight non‐exclusive categories that reflected a multiplicity of neurochemical MOAs (Roth, Sheffler, & Kroeze, 2004). These are presented in Data S1. Adherence to medications was confirmed by patient report and verified by collateral history from their prescribing physicians.

**Table 1:** Medications categorized by standard classes

Standard category	Medication name
SSRI	Citalopram (Celexa)
	Escitalopram (Lexapro)
	Fluoxetine (Prozac)
	Fluvoxamine (Luvox)
	Paroxetine (Paxil)
	Sertraline (Zoloft)
	Vilazodone (Viibryd)
SNRI	Desvenlafaxine (Pristiq)
	Duloxetine (Cymbalta)
	Levomilnacipran (Fetzima)
	Venlafaxine (Effexor XR)
TCA	Amitriptyline (Elavil)
	Clomipramine
	Desipramine (Norpramin)
	Doxepin
	Imipramine
	Nortriptyline (Pamelor)
MAOI	Phenelzine
	Selegiline (Emsam)
	Tranylcypromine (Parnate)
Atypical Antidepressant	Bupropion (Wellbutrin/Wellbutrin SR)
	Mirtazapine (Remeron)
	Nefazodone (Serzone)
	Trazodone (Oleptro)
	Vortioxetine (Brintellix)
Atypical Antipsychotic	Aripiprazole (Abilify)
	Asenapine (Saphris)
	Lurasidone (Latuda)
	Olanzapine (Zyprexa)
	Quetiapine (Seroquel)
	Risperidone (Risperdal)
	Ziprasidone (Geodon)
Typical Antipsychotic	Haloperidol (Haldol)
Anti‐Epileptic	Carbamazepine (Tegretol)
	Gabapentin (Neurontin)
	Lamotrigine (Lamictal)
	Oxcarbazepine
	Pregabalin (Lyrica)
	Primidone (Mysoline)
	Topiramate (Topamax)
	Valproic acid (Depakote)
Benzodiazepine	Alprazolam (Xanax/Niravam)
	Chlordiazepoxide (Librium)
	Clonazepam (Klonopin)
	Diazepam (Valium)
	Lorazepam (Ativan)
	Temazepam (Restoril)
	Triazolam
	Quazepam (Doral)
Quasi‐Benzodiazepine	Eszopiclone
	Zolpidem
Psychostimulant	Amphetamine/Dextroamphetamine (Adderall)
	Armodafinil (Nuvigil)
	Dexmethylphenidate (Focalin)
	Dextroamphetamine (Dexedrine)
	Lisdexamfetamine (Vyvanse)
	Methylphenidate (Concerta, Ritalin, Methylin)
	Modafinil (Provigil)
Lithium	Lithium
Other	Amlodipine
	Baclofen
	Buspirone
	Clonidine
	Ephedrine
	Prazosin
	Propranolol
	Tizanidine
	Verapamil

### 2.3 Subjects’ medication coding

Medication information for each subject was obtained from electronic records that listed all medications at the beginning of treatment. Subject data were coded in a binary “yes/no” fashion for each category. For example, a subject taking only venlafaxine, would have been coded as “1” under the standard category “SNRI.” For medications having dose‐dependent effects, each subject was coded as warranted by the dose. For example, the following medications were considered largely sub‐therapeutic and therefore lacking significant effects at their primary targets, at the following daily doses: trazodone (below 100 mg); quetiapine (below 100 mg); venlafaxine (below 100 mg); mirtazapine (below 15 mg). For psychotropic medications used on an as‐needed (prn) basis, we estimated the average daily dose by patient report and then used our standard criteria. For those rare patients who started or stopped a medication during the course of rTMS (approximately 5%), we included them in that category as a conservative measure. For each subject, we computed “Total number of medications,” to serve as general measure of overall medication use or burden.

### 2.4 rTMS treatment

Subjects were treated using the NeuroStar TMS System (Neuronetics, Inc, Malvern, PA) with 30 sessions scheduled over six weeks. Treatment began using parameters of 3,000 pulses per session at 10 Hz administered to the left dorsolateral prefrontal cortex (DLPFC) with a 40‐pulse train and intertrain interval of 26 s (total duration 37.5 min). Intensity was titrated up to 120% of the resting motor threshold (MT) as tolerated. After the first two weeks, “flexible‐dosing” adjustment options included: increasing the number of 10 Hz pulses delivered to the left DLPFC; sequential bilateral treatment (Fitzgerald et al., 2006) with addition of right‐sided 1 Hz stimulation targeting the right DLPFC; or switching to 1 Hz stimulation of right DLPFC in the absence of benefit from bilateral stimulation (McDonald et al., 2011). Within these options, up to 5,000 total pulses were delivered per session. Treatment adjustments were guided by changes in symptom severity, and physician clinical judgment, within established treatment guidelines utilizing a “measurement‐based care” approach (Guo et al., 2015).

### 2.5 Data analysis

Medication categories that were in use by at least 20% of the sample were examined in association with clinical outcomes. This strategy reduced the number of tests and ensured meaningfully sized analysis samples. Change in the IDS‐SR30 total score from baseline to week 2, and change in the IDS‐SR30 total score across weeks 2, 4, and 6, were examined as co‐primary outcomes, using linear regression and linear mixed model analysis, respectively. Analyses were performed using SPSS version 24; because this was an exploratory, hypothesis‐generating investigation, we reported all findings meeting a significance threshold of *p* ≤ 0.05 without correction for multiple comparisons.

The week 2 outcome was of specific interest because the first 2 weeks were the most homogeneous with respect to treatment parameters including intensity (100%–120% MT), “dose” (~3,000 pulses), frequency (10 Hz), and site (i.e., left DLPFC). Separate linear regression analyses were performed to examine each medication category as a dichotomous predictor of raw change in the IDS‐SR30 total score at week 2, in models that included the baseline IDS‐SR30 total score as a covariate. Medication categories that were identified as significant predictors after controlling for overall baseline severity were then evaluated in further models that examined baseline IDS‐SR30 anxiety and non‐anxiety item totals as separate covariates, and examined covariates of age and other clinical characteristics (i.e., total number of medications) that differed between users and non‐users of a given medication category. Baseline anxiety was assessed using an 8‐item subscale (IDS‐SR30 items 6, 23, 24, 25, 26, 27, 28, 30) (Wardenaar et al., 2010). In statistical models that examined baseline anxiety as a covariate, IDS‐SR30 item totals were parsed into an anxiety subscale, and a non‐anxiety subscale in order to avoid collinearity between the subscale and the total score.

Linear mixed model analyses were used to assess relationships between each medication category and change in IDS‐SR30 total score over 6 weeks of treatment. This approach was used to examine associations between medication use and response to clinical rTMS more generally, that is, as a treatment modality, allowing for variability and flexible changes in specific parameters. We compared symptom severity changes between users versus non‐users of each medication category in separate linear mixed model analyses conducted using restricted maximum likelihood (REML). Changes in IDS‐SR30 total score at weeks 2, 4, and 6 were calculated from baseline, yielding a within‐group factor of time with three levels. Mixed models examined change over time, co‐varying for baseline IDS‐SR30. Other covariates were examined as dictated by the specific medication category.

## 3 RESULTS

### 3.1 Clinical, demographic, and medication use characteristics of the sample

The analyzable sample (*n* = 181) included 98 females and 83 males with a mean age of 46.6 ± 16.6 years entering treatment with a mean IDS‐SR30 total score of 42.8 ± 11.1, and an anxiety subscale score of 9.7 ± 4.3. Overall, 92% of subjects were taking at least one psychotropic medication; 78% were taking at least one antidepressant. Among medication users, the mean number of medications was 4.2 ± 2.4, including 1.3 ± 0.8 antidepressants. Table 2 shows medication use by category. Seven of twelve clinically‐based medication categories were in use by 20% or more of the sample.

**Table 2:** Numbers and percentages of patients taking medications during acute rTMS treatment for depression, grouped by standard non‐exclusive categories

Medication category	Number of patients taking medication	Proportion (%) of the sample (*n* = 181)
SSRI	62	34.3
SNRI	42	23.2
TCA	9	5.0
MAOI	12	6.6
Atypical Antidepressant	73	40.3
Atypical Antipsychotic	56	30.9
Typical Antipsychotic	1	0.6
Anti‐Epileptic	58	32.0
Benzodiazepine	72	39.8
Quasi‐benzodiazepine	16	8.8
Psychostimulant	56	30.9
Lithium	15	8.3
Other	30	16.6

### 3.2 Clinical outcomes in the overall sample

Subjects showed a mean decrease (improvement) of 7.9 ± 9.8 points on the IDS‐SR after 2 weeks of treatment. Change in symptom severity at week 2 was not associated with gender, baseline IDS‐SR30, baseline anxiety, or total number of psychotropic or antidepressant medications, but was significantly associated with age (*p* = 0.04) where older subjects showed greater improvement. After 6 weeks of treatment, subjects improved by 13.8 ± 12.1 points. Week 6 improvement was associated with baseline IDS‐SR30 total (*r* = −0.27, *p* = 0.001) and anxiety subscale (*r* = −0.16, *p* = 0.05) scores, with higher baseline scores associated with greater decreases. 47% of those who received solely left‐sided treatment responded to treatment at week 6, versus 19% of those who had right‐side stimulation added at some point during their treatment course (*χ* ^2^ = 13.386, *p* < 0.001). Those patients who received right‐sided stimulation added also showed significantly smaller decreases in the IDS score total than those who had left‐sided stimulation only (*t* = 3.033, *p* = 0.003).

### 3.3 Medication categories and week 2 outcome

Regression models for each medication category, controlling for baseline IDS‐SR30, found significant effects for BDZs (*p* = 0.02) and psychostimulants (*p* = 0.05) (Table 3a). BDZ use was associated with less improvement at week 2, whereas psychostimulant use was associated with greater improvement. These medication effects remained significant after adding covariates of age, baseline anxiety, and total number of medications (Table 3b). Age was examined as a covariate because it had been significantly associated with week 2 outcome, and anxiety because it is often associated with BDZ use. Furthermore, baseline anxiety was greater in psychostimulant users versus non‐users (10.8 ± 4.4 vs. 9.2 ± 4.1, *p* = 0.02). Total number of medications used was greater in psychostimulant users versus non‐users (4.8 ± 2.4 vs. 3.4 ± 2.6; *p* < 0.001).

**Table 3:** Results of linear regression analyses examining medication categories as predictors of week 2 outcome of rTMS for depression: (a) Models with baseline IDS‐SR30 total score covariate only; (b) Significant models with baseline anxiety and non‐anxiety item total covariates, and additional covariates^1^

Medication category	Overall model	Baseline IDS *p*‐value	Medication category *p*‐value
Benzodiazepine	*F* = 4.36; *p *= 0.014	0.05	0.02*
Psychostimulant	*F* = 3.45; *p *= 0.034	0.12	0.05*
SSRI	*F* = 1.41; *p *= 0.247	0.10	0.92
SNRI	*F* = 1.44; *p *= 0.240	0.09	0.80
Atypical Antidepressant	*F* = 1.65; *p *= 0.195	0.09	0.49
Atypical Antipsychotic	*F* = 1.68; *p *= 0.190	0.10	0.47
Anti‐Epileptic	*F* = 1.41; *p *= 0.246	0.10	0.89

Medication category	Model Sig.	*R* ^2^	Covariates and significance	Medication category statistics			
				Unstandardized β	Std. Error	*t*	*p*
Benzodiazepine	0.008	0.056	Baseline non‐anxiety IDS (*p* = 0.019)	3.42	1.52	2.25	0.03*
	0.010	0.066	Baseline non‐anxiety IDS (*p* = 0.007), Baseline anxiety (*p* = 0.182, N.S.)	3.22	1.52	2.11	0.04*
	0.005	0.087	Baseline non‐anxiety IDS (*p* = 0.009), Baseline anxiety (*p* = 0.386, N.S.), Age (*p* = 0.053, N.S.)	3.33	1.51	2.20	0.03*
	0.002	0.083	Baseline non‐anxiety IDS (*p* = 0.011), Age (*p* = 0.028)	3.47	1.50	2.31	0.02*
Psychostimulants	0.020	0.046	Baseline non‐anxiety IDS (*p* = 0.034)	−2.93	1.60	−1.83	0.07
	0.006	0.083	Baseline non‐anxiety IDS (*p* = 0.004), Baseline Anxiety (*p* = 0.068), Total Meds (*p* = 0.093)	−4.15	1.65	−2.51	0.01**
	0.002	0.111	Baseline IDS (*p* = 0.004), Baseline Anxiety (*p* = 0.198, N.S.), Total Meds (*p* = 0.040), Age (*p* = 0.023)	−4.44	1.64	−2.71	0.007**

*p ≤ 0.05; **p ≤ 0.01.

### 3.4 Medication categories and changes in symptom severity over weeks 2, 4, 6

**Figure 2:**
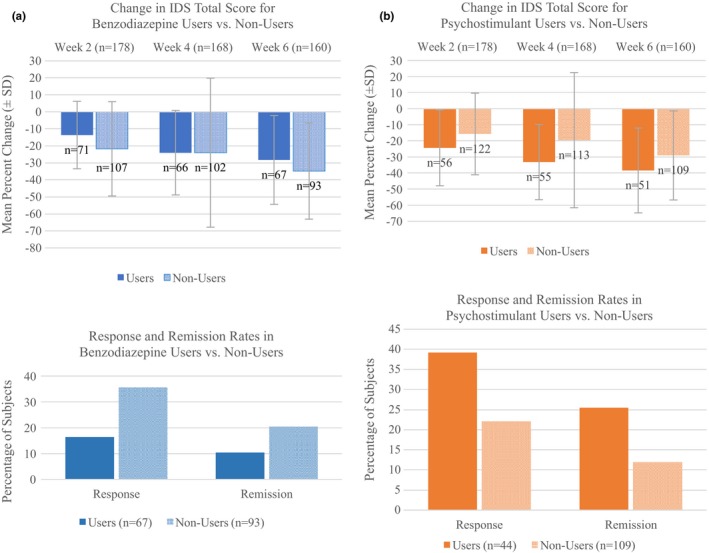
Change in IDS total score (mean and standard deviation) at weeks 2, 4, 6 and rates of response and remission, for users versus non‐users of benzodiazepines (a) and psychostimulants (b)

Psychostimulant use was significantly associated with greater improvement over the course of treatment including baseline IDS‐SR30 as a covariate (*F*_(1, 176.744) _= 4.94, *p* = 0.03) (Table 4). Estimated marginal means were −13.09 for subjects who were taking a psychostimulant versus −9.56 for those who were not. Total number of medications and anxiety subscale variables were not significant in any of the mixed models and so were excluded from final models. The interaction between time and medication category was not significant in any of the models. Figure 2 shows IDS‐SR30 changes for benzodiazepine and psychostimulant users and non‐users at weeks 2, 4, and 6.

**Table 4:** Medication effects in linear mixed model analyses examining clinically based medication categories as predictors of change in symptom severity over time (weeks 2, 4, and 6)^2^

	Denominator *df*	*F*	*p*
Psychostimulants	176.74	4.94	0.03*
Benzodiazepine	178.06	3.00	0.09
SSRI	177.48	0.00	0.99
SNRI	177.30	0.00	0.99
Atypical Antidepressant	177.45	0.11	0.74
Atypical Antipsychotic	177.43	0.03	0.86
Anti‐Epileptic	177.24	0.28	0.60

Note

All models included baseline severity as a covariate. Change in symptom severity was assessed using the Inventory of Depressive Symptomatology Self Report (IDS‐SR30) (Rush et al., 1996).

* *p* ≤ 0.05.

Considering a response criterion of 50% improvement in the IDS‐SR30 total score at week 6, the response rate was lower in BDZ‐users versus BDZ‐non‐users (16.4% vs. 35.5%, *p* = 0.008), and higher in psychostimulant users versus non‐users (39.2% vs. 22.0%, *p* = 0.02).

### 3.5 Medication categories in relation to rTMS treatment parameters

Chi‐square analysis showed no significant difference between those who received solely 10 Hz left rTMS treatment compared to those who received 1 Hz right sided treatment in terms of the frequency of benzodiazepine (*χ* ^2^ = 1.206, *p* = 0.33) or psychostimulant use (*χ* ^2^ = 1.264, *p* = 0.40).

## 4 DISCUSSION

This exploratory observational study found that benzodiazepine use was associated with less improvement, whereas psychostimulant use was associated with greater improvement, after two weeks of rTMS treatment for depression. The relationships between medication use and clinical outcome were statistically significant even when controlling for baseline age, symptom severity, and severity of anxiety symptoms (*p* < 0.05 without correction for multiple comparisons). The week two results are of interest because most subjects (78%) had received standardized 10 Hz rTMS targeting left DLPFC only. Across the entire six weeks of treatment, psychostimulant use was associated with greater improvement, again after controlling for baseline variables. Treatment over the entire six‐week course involved greater variability; by week 6, 1 Hz rTMS targeting right DLPFC had been introduced into treatment for 70% of the sample. Given that psychostimulant effects were observed at week 2 and across six weeks of treatment, this medication category may be more likely to be associated with rTMS outcome for depression regardless of treatment duration, or the site/frequency of stimulation.

The present results suggest that BDZ use is associated with less improvement early in the course of rTMS. (Supplementary analyses showed that a broader group of GABA agonists, which included BDZ, was associated with poorer six‐week outcome. See Data S1). There is evidence of disturbances in the GABAergic system in MDD, including decreased cortical concentrations of GABA, as measured by magnetic resonance spectroscopy (MRS) (Pehrson & Sanchez, 2015), as well as emerging evidence that some effective treatments for depression are associated with enhanced or remediated GABA function. One prior rTMS investigation reported a trend‐level *negative* association between change in medial prefrontal cortex GABA during a course of TMS and daily use of lorazepam (Dubin et al., 2016). In rodent models, long‐term administration of BDZ is associated with down‐regulatory effects on GABA signaling, such as decreases in GABA_A_ receptor subunit expression, including cortical alpha‐1 mRNA (Uusi‐Oukari & Korpi, 2010), decreased alpha‐1 polypeptide (Chen, Huang, Zeng, Sieghart, & Tietz, 1999; Impagnatiello et al., 1996; Pesold et al., 1997), and modified surface dynamics of GABA_A_Rs (Gouzer, Specht, Allain, Shinoe, & Triller, 2014). These effects suggest that the concurrent, chronic use of BDZ medications could tend to mitigate the probable increases in cortical GABA signaling that appear with clinically effective rTMS.

Psychostimulants (in use by 31% of our sample) could enhance rTMS treatment outcomes through enhancement of plasticity as catecholaminergic agonists, most likely acting through adrenergic pathways. NE is a strong modulator of cortical plasticity, for example, in the hippocampus (Gu, 2002). This finding also is consistent with the results of experiments testing the effects of noradrenergic agents on single‐ and paired‐pulse TMS paradigms. Catecholamine transport inhibitors such as methylphenidate enhance the practice effect on single‐pulse TMS‐induced movement (Meintzschel & Ziemann, 2005), and amphetamine enhances practice‐related changes in cortical motor mapping (Tegenthoff, Cornelius, Pleger, Malin, & Schwenkreis, 2004). Conversely, the selective alpha‐1 adrenergic receptor antagonist prazosin abolishes the LTP‐like motor‐evoked potential response in the paired‐associate TMS paradigm (Korchounov & Ziemann, 2011). These effects on plasticity are not mediated by changes in excitability per se*, *because the motor threshold is not altered by methylphenidate (Gilbert et al., 2006), amphetamine (Boroojerdi, Battaglia, Muellbacher, & Cohen, 2001; Ziemann, Tergau, Bruns, Baudewig, & Paulus, 1997), atomoxetine (Gilbert et al., 2006), guanfacine (Boroojerdi et al., 2001), or yohimbine (Plewnia, Bartels, Cohen, & Gerloff, 2001). It is possible that these plasticity‐modulating effects also are synergistic with rTMS, giving rise to relatively enhanced clinical outcomes for those patients concurrently taking noradrenergic agonist agents.

The present results could be consistent with an interaction between stimulation site/frequency and medication, in which benzodiazepines counter and psychostimulants potentiate the effects of left‐sided fast rTMS. Conversely, these medications might have opposite effects in the context of right‐sided slow rTMS. This would be supported by the finding that benzodiazepine use is associated with less clinical benefit in the first two weeks of treatment only, when most patients received only left‐sided stimulation. Because treatment was not assigned in a controlled manner in this study, we cannot definitively address this question. Future work is needed to examine medication use and rTMS treatment outcomes in controlled experimental treatment.

We found no significant interaction between the administration of antipsychotic medication and rTMS treatment outcome in these patients. These findings are consistent with the prior work of Hu and colleagues (2016), who reported no benefit from concomitant quetiapine administration in Bipolar II depressed patients receiving rTMS, though only partially consistent with the prior report of Schulze and colleagues (2017), who found a non‐significant trend towards greater improvement among patients receiving theta‐burst rTMS and antipsychotic medication. Future studies should examine the interactions of class of medications with different rTMS treatment parameters.

### 4.1 Limitations

This study is significant in that it offers the first systematic survey of relationships among the use of commonly prescribed medications and rTMS treatment outcome in MDD. Strengths of the study are the large number of subjects examined and the broad range of medications considered. Nevertheless, results should be interpreted in the context of certain limitations. First, this study was observational and did not utilize controlled assignment to type(s) of medication or rTMS treatment. There were significant differences in outcome of those receiving exclusively left‐sided versus right‐sided stimulation at some point in the course of their rTMS treatment, although there were no significant differences in benzodiazepine or stimulant use between the two treatment groups. Subjects entered the study taking medications that were prescribed on clinical grounds by prior treating physicians. These data suggest that groups of subjects receiving different rTMS treatment paradigms and/or medications may have had underlying neurobiological or clinical differences that could account in part for the observed differences in outcome, apart from the effects of treatment alone. We did not have diagnostic information on possible comorbid psychiatric diagnoses, nor a measure of the degree of treatment; either comorbid diagnoses or differences in treatment resistance could contribute to differences in treatment outcome. Second, a major caveat is that we explored relationships between a large number of medication categories without correction for multiple comparisons. These results should therefore be considered as hypothesis‐generating. These results are novel and indicate the importance of future experimental studies of drug/TMS interactions, to address both neurobiological and clinical effects. It remains premature to make clinical recommendations on the basis of the foregoing evidence; however, this represents a future goal of this work. Third, the absence of significant results for the less‐often prescribed medication categories may represent false‐negative results. Although we constrained analyses to those categories that were prescribed to at least 20% of the sample, analyses may have been underpowered to detect effects. Fourth, these results do not account for possible nonlinear effects of medications, or possible complex pharmacodynamic effects in subjects receiving medication combinations; therefore, results may reflect interactions among simultaneous medications. Lastly, we had no information on differences among subjects in their rates of metabolism for the drugs prescribed and limited information on medication adherence, which made it impossible to compare pharmacodynamic and pharmacokinetic effects across the sample.

## 5 CONCLUSION

The findings of this manuscript indicate important lines for future research. It is possible that concomitant medication use may, depending on the drug category or MOA, impact rTMS treatment outcome favorably or unfavorably. The present findings, if replicated with appropriate controls, could have implications for the concurrent use of psychotropic medications during rTMS treatment. Prospective hypothesis‐based studies controlling for multiple medications and treatment‐resistance are needed to guide decisions about use of common medications during rTMS treatment.

## DISCLOSURES

Dr. Minzenberg owns stock in Elan Pharmaceuticals and has received investigator‐initiated support from Shire. Dr. Cook reports he has received research support from Covidien (formerly Aspect Medical Systems), National Institutes of Health, and NeoSync, Inc. within the past three years; he has been an advisor/consultant/reviewer for Arctica Health, Cerêve, HeartCloud, NeuroDetect, NeuroSigma, NIH (ITVA), U.S. Departments of Defense and Justice, and the VA (DSMB); he is editor of the Patient Management section of the American Psychiatric Association's FOCUS journal; his biomedical intellectual property is assigned to the Regents of the University of California, and he has stock options in NeuroSigma, where he has served as Chief Medical Officer (on leave); he is employed by the University of California, Los Angeles and also has an appointment as a Staff Psychiatrist, Neuromodulation and Mood Disorders programs, Greater Los Angeles Veterans Administration Health System. Dr. Krantz discloses that within the past 36 months he has received research support from the National Institutes of Health. He currently serves as an investigator on a study sponsored by Neosync, Inc. Dr. Leuchter discloses that within the past 36 months he has received research support from the National Institutes of Health, Neuronetics, Department of Defense, CHDI Foundation, and NeuroSigma, Inc. He has served as a consultant to NeoSync, Inc., Ionis Pharmaceuticals, Inc., and ElMindA. He is Chief Scientific Officer of Brain Biomarker Analytics LLC (BBA). Dr. Leuchter owns stock options in NeoSync, Inc. and has equity interest in BBA. Dr. Levitt, Dr. Hunter, Ms. Rotstein and Ms. Chawla do not have anything to disclose.

## AUTHOR CONTRIBUTION

Drs. Hunter and Minzenberg contributed equally to this work.

## Footnotes

1 [Correction added on 17 April 2019, after first online publication: ‘Standard category’ was removed as a sub‐header under Medication category.]

2 [Correction added on 17 April 2019, after first online publication: the following changes have been made (1) MOA‐based medical categories are not included and this has been deleted in the table caption; and (2) ‘Standard category’ was removed as a sub‐header under Medication category.]
