# Supplementary material for: Concomitant medication use and clinical outcome of repetitive Transcranial Magnetic Stimulation (rTMS) treatment of Major Depressive Disorder
Source: Brain Behav. 2019 Apr 2;9(5):e01275. doi: 10.1002/brb3.1275 (PMC6520297; doi:10.1002/brb3.1275)
Supplement: Supplementary file 1 [file BRB3-9-e01275-s001.docx]

**Supporting Information**

In addition to the use of standard medication categories, we examined the effects of medication on outcome using a novel mechanism of action (MOA) based schema based on the neurochemical actions of individual drugs. The standard taxonomy is most relevant to a treatment-oriented perspective, whereas the MOA-based categories (cutting across conventional, clinically-defined classes) could help advance our understanding of how neurochemical systems mediate or modify rTMS therapeutic effects. The medication categories and medications are detailed in Supporting Table 1**.** These categories included: Catecholaminergic agonist (CA_Ag); Catecholaminergic antagonist (CA_Ant); Serotoninergic agonists (5HT_Ag); Primarily 5HT action (Mostly5HT, e.g. SSRIs); 5HT and norepinephrine (NE) action (5HT&NE, e.g. SNRIs, most MAOIs, some TCAs); Primarily NE action (MostlyNE, e.g. stimulants); Voltage-gated ion channel blocker (IonChBlocker, e.g. most antiepileptic drugs); and GABA agonist (GABA_Ag, e.g., direct or indirect, including BDZs, quasi-BDZ sleep agents, baclofen, and some AEDs). Drugs were categorized as primarily affecting a neurotransmitter system (e.g., “MostlyNE”) if they exhibit in vitro binding at an element of one neurotransmitter system with affinity at least 10-fold higher than a second neurotransmitter system, while they were listed as mixed (e.g., 5HT&NE) if they had binding affinities within one order of magnitude of each other throughout most of the dose range (e.g., duloxetine, nortriptyline) (Connolly & Thase, 2016; Fišar, 2016; Minzenberg, Poole, Benton, & Vinogradov, 2004; Miyamoto, Duncan, Marx, & Lieberman, 2005; Rogawki & Löscher, 2004; Tatsumi, Groshan, Blakely, & Richelson, 1997; Tatsumi, Jansen, Blakely, & Richelson, 1999; Vaishnavi et al., 2004). Agonist and antagonist categories identify functional effects, not strictly direct receptor-binding effects. For example, mirtazapine is a functional agonist of NE and 5HT (i.e., as an antagonist of both NE and 5HT autoreceptors, it augments release of both NE and 5HT). Conversely, guanfacine and clonidine are functional *ant*agonists (in this case, of NE), as they act as agonists of the NE autoreceptor, thereby decreasing NE release, or on post-synaptic NE receptors (Arnsten & Jin, 2014). For both mirtazapine and the alpha-2 adrenergic agonists, the net effects mediated via these autoreceptors or adrenergic receptors represent the basis for most or all of their clinical effects. Aripiprazole is a special case among psychotropic drugs used in depression; it is defined here as a functional catecholamine agonist (CA_Ag), as this best-describes its actions at D2 receptors, especially in conditions where background dopamine (DA) release is not elevated (Gründer, Carlsson, & Wong, 2003).

We aimed to be exhaustive in labelling all medications by as many labels as justified by the basic pharmacology literature without exaggerating the effects of different MOAs at therapeutic dosages. For example, ziprasidone is reasonably categorized not only as “CA_Ant”, but also as “NE&5HT” given its strong affinity for both the NET and SERT (Tatsumi et al., 1999).

Subject data were coded in a binary ‘yes/no’ fashion for each MOA-based category. For example, a subject taking only venlafaxine, would have been coded as ‘1’ under the standard category ‘SNRI’, and coded as ‘1’ under each of three MOA categories: ‘CA_Ag,’ ‘5HT_Ag,’ and ‘Mostly5HT.’ For medications having dose-dependent effects, each subject was coded for the appropriate MOA-based categories as warranted by the dose. For example, the following medications were considered largely sub-therapeutic and therefore lacking significant effects at their primary targets, at the following daily doses: trazodone (below 100 mg); quetiapine (below 100 mg); venlafaxine (below 100 mg); mirtazapine (below 15 mg). For psychotropic medications used on an as-needed (prn) basis, we estimated the average daily dose by patient report and then used our standard criteria. For those rare patients who started or stopped a medication during the course of rTMS (approximately 5%), we included them in that category as a conservative measure.

**Results**

**MOA categories and week 2 outcome**

Each of the eight MOA-based categories was used by at least 20% of the sample (Supporting Table 2). Regression models for each medication category, controlling for baseline IDS-SR30, found significant effects for GABA_Ag (p = 0.01), MostlyNE (p = 0.04), and CA_Ant MOA (p = 0.01) categories (Supporting Table 3A). GABA_Ag and CA_Ant were associated with less improvement at week 2, whereas MostlyNE were associated with greater improvement. Medication effects for each of these categories remained significant after adding covariates of age, baseline anxiety, and total number of medications (Supporting Table 3B). Age was examined as a covariate because it had been significantly associated with week 2 outcome. Anxiety was examined as a covariate because it is often associated with GABA_Ag use. Furthermore, baseline anxiety was greater in GABA_Ag users vs. non-users (10.59±4.45 vs. 8.76±3.90, p < 0.001). Total number of medications used was greater in GABA_Ag users vs. non-users (4.85±2.74 vs. 2.78±1.98; p < 0.001), and in CA_Ant users vs. non-users (5.5±3.2 vs. 3.5±2.3; p < 0.001). Additionally, because baseline analyses showed a significantly greater number of antidepressant medications for MostlyNE users vs. non-users (1.42±0.77 vs. 0.90±0.84, p < 0.001), this variable was examined as a covariate in MostlyNE models; it was not a significant predictor.

Chi square analyses did not show any significant difference in the rates at which patients receiving exclusively left-sided vs. right-sided rTMS at week 2 were using catecholamine antagonists (χ^2^ = 1.604, p = .20), mostly-NE medications (χ^2^ = 1.686, p = .19), or GABA agonists (χ^2^ = 0.350, p = .55).

**MOA categories and changes in symptom severity over weeks 2, 4, 6**

The MostlyNE category was significantly associated with greater improvement over the course of acute treatment, whereas the GABA_Ag category was associated with less improvement. All models included baseline IDS-SR30 as a covariate (Supporting Table 4). MostlyNE use was significant with F_(1, 178.615)_ = 4.14, p = 0.04. Estimated marginal means were -12.07 for those taking a MostlyNE medication vs. -9.07 for those who were not. GABA_Ag use was significant with F_(1,178.188)_ = 4.14, p = 0.04. GABA_Ag estimated marginal means for users vs. non-users were -9.23 vs. -12.25. Total number of medications and anxiety subscale variables were not significant in any of the mixed models and so were excluded from final models. The interaction between time and medication category was not significant in any of the models.

Chi square analyses did not show any significant difference in the rates at which patients receiving exclusively left-sided vs. right-sided rTMS over the course of treatment were using catecholamine antagonists (χ^2^ = 0.536, p = .46), mostly-NE medications (χ^2^ = 1.300, p = .25), or GABA agonists (χ^2^ = 1.086, p = .30).

**Discussion**

These supplementary data analyses showed that GABA agonist and catecholamine antagonist medication use were associated with less improvement after two weeks of rTMS treatment for depression. The relationships between medication use and clinical outcome were statistically significant (p < .05 without correction for multiple comparisons) even when controlling for baseline age, symptom severity, and severity of anxiety symptoms. The week two results are of interest because most subjects (78%) had received standardized 10 Hz rTMS targeting left DLPFC only. Across the entire six weeks of treatment, GABA agonists (including benzodiazepines) were again associated with less improvement, whereas medications having mostly norepinephrine MOA were associated with greater improvement. These effects again were significant over and above other baseline explanatory variables including anxiety. Treatment over the entire six-week course involved greater variability; by week 6, 1Hz rTMS targeting right DLPFC had been introduced into treatment for 70% of the sample. Given that GABA agonist were observed at week 2 and across six weeks of treatment, these medication categories may be associated with rTMS outcome for depression regardless of treatment duration, or the site/frequency of stimulation.

**References**

Arnsten, A. F., & Jin, L. E. (2014). Molecular influences on working memory circuits in

dorsolateral prefrontal cortex. *Progress in molecular biology and translational science, 122*, 211-231. Academic Press. https://doi.org/10.1016/B978-0-12-420170-5.00008-8

Connolly, K. R., & Thase, M. E. (2016). Vortioxetine: a new treatment for major

depressive disorder. *Expert opinion on pharmacotherapy*, *17*(3), 421-431. https://doi.org/10.1517/14656566.2016.1133588

Fišar, Z. (2016). Drugs related to monoamine oxidase activity. *Progress in Neuro-*

*Psychopharmacology and Biological Psychiatry*, *69*, 112-124. https://doi.org/10.1016/j.pnpbp.2016.02.012

Gründer, G., Carlsson, A., & Wong, D. F. (2003). Mechanism of new antipsychotic

medications: occupancy is not just antagonism. *Archives of general psychiatry*, *60*(10), 974-977. https://doi.org/10.1001/archpsyc.60.10.974

Minzenberg, M. J., Poole, J. H., Benton, C., & Vinogradov, S. (2004). Association of

anticholinergic load with impairment of complex attention and memory in schizophrenia. *American Journal of Psychiatry*, *161*(1), 116-124. https://doi.org/10.1176/appi.ajp.161.1.116

Miyamoto, S., Duncan, G. E., Marx, C. E., & Lieberman, J. A. (2005). Treatments for

schizophrenia: a critical review of pharmacology and mechanisms of action of antipsychotic drugs. *Molecular psychiatry*, *10*(1), 79-104. https://doi.org/10.1038/sj.mp.4001556

Rogawski, M. A., & Löscher, W. (2004). The neurobiology of antiepileptic drugs. *Nature*

*Reviews Neuroscience*, *5*(7), 553- 564. https://doi.org/10.1038/nrn1430

Rush, A. J., Gullion, C. M., Basco, M. R., Jarrett, R. B., & Trivedi, M. H. (1996). The inventory

of depressive symptomatology (IDS): psychometric properties. *Psychological medicine*,

*26*(3), 477-486.

Tatsumi, M., Groshan, K., Blakely, R. D., & Richelson, E. (1997). Pharmacological profile

of antidepressants and related compounds at human monoamine transporters. *European journal of pharmacology*, *340*(2-3), 249-258. https://doi.org/10.1016/S0014-2999(97)01393-9

Tatsumi, M., Jansen, K., Blakely, R. D., & Richelson, E. (1999). Pharmacological profile

of neuroleptics at human monoamine transporters. *European journal of pharmacology*, *368*(2-3), 277-283. https://doi.org/10.1016/S0014-2999(99)00005-9

Vaishnavi, S. N., Nemeroff, C. B., Plott, S. J., Rao, S. G., Kranzler, J., & Owens, M. J.

(2004). Milnacipran: a comparative analysis of human monoamine uptake and

transporter binding affinity. *Biological psychiatry*, *55*(3), 320-322. https://doi.org/10.1016/j.biopsych.2003.07.006

**Supporting Tables**

**Supporting Table 1. Medications categorized by MOA–based schema.**

| **Standard Category** | **Medication Name** | **MOAs** | | | |
| --- | --- | --- | --- | --- | --- |
| SSRI | Citalopram (Celexa) | 5HT_Ag | Mostly5HT |  |  |
|  | Escitalopram (Lexapro) | 5HT_Ag | Mostly5HT |  |  |
|  | Fluoxetine (Prozac) | 5HT_Ag | Mostly5HT |  |  |
|  | Fluvoxamine (Luvox) | 5HT_Ag | Mostly5HT |  |  |
|  | Paroxetine (Paxil) | CA_Ag | 5HT_Ag | Mostly5HT |  |
|  | Sertraline (Zoloft) | 5HT_Ag | Mostly5HT |  |  |
|  | Vilazodone (Viibryd) | 5HT_Ag | Mostly5HT |  |  |
| SNRI | Desvenlafaxine (Pristiq) | CA_Ag | 5HT_Ag | Mostly5HT |  |
|  | Duloxetine (Cymbalta) | CA_Ag | 5HT_Ag | 5HT&NE |  |
|  | Levomilnacipran (Fetzima) | CA_Ag | 5HT_Ag | 5HT&NE |  |
|  | Venlafaxine (Effexor XR) | CA_Ag | 5HT_Ag | Mostly5HT |  |
| TCA | Amitriptyline (Elavil) | CA_Ag | 5HT_Ag | 5HT&NE |  |
|  | Clomipramine | CA_Ag | 5HT_Ag | Mostly5HT |  |
|  | Desipramine (Norpramin) | CA_Ag | 5HT_Ag | MostlyNE |  |
|  | Doxepin | CA_Ag | 5HT_Ag | 5HT&NE |  |
|  | Imipramine | CA_Ag | 5HT_Ag | 5HT&NE |  |
|  | Nortriptyline (Pamelor) | CA_Ag | 5HT_Ag | 5HT&NE |  |
| MAOI | Phenelzine | CA_Ag | 5HT_Ag | 5HT&NE |  |
|  | Selegiline (Emsam) | CA_Ag | MostlyNE |  |  |
|  | Tranylcypromine (Parnate) | CA_Ag | 5HT_Ag | 5HT&NE |  |
| Atypical Antidepressant | Bupropion (Wellbutrin /  Wellbutrin SR) | CA_Ag | MostlyNE |  |  |
|  | Mirtazapine (Remeron) | CA_Ag | 5HT_Ag | 5HT&NE |  |
|  | Nefazodone (Serzone) | CA_Ag | 5HT_Ag | 5HT&NE |  |
|  | Trazodone (Oleptro) | 5HT_Ag | Mostly5HT |  |  |
|  | Vortioxetine (Brintellix) | 5HT_Ag | Mostly5HT |  |  |
| Atypical Antipsychotic | Aripiprazole (Abilify) | CA_Ag |  |  |  |
|  | Asenapine (Saphris) | CA_Ant |  |  |  |
|  | Lurasidone (Latuda) | CA_Ant |  |  |  |
|  | Olanzapine (Zyprexa) | CA_Ant |  |  |  |
|  | Quetiapine (Seroquel) | CA_Ant |  |  |  |
|  | Risperidone (Risperdal) | CA_Ant |  |  |  |
|  | Ziprasidone (Geodon) | CA_Ag | CA_Ant | 5HT_Ag | 5HT&NE |
| Typical Antipsychotic | Haloperidol (Haldol) | CA_Ant |  |  |  |
| Anti-Epileptic | Carbamazepine (Tegretol) | IonChBlocker |  |  |  |
|  | Gabapentin (Neurontin) | IonChBlocker | GABA_Ag |  |  |
|  | Lamotrigine (Lamictal) | IonChBlocker |  |  |  |
|  | Oxcarbazepine | IonChBlocker |  |  |  |
|  | Pregabalin (Lyrica) | IonChBlocker | GABA_Ag |  |  |
|  | Primidone (Mysoline) | GABA_Ag |  |  |  |
|  | Topiramate (Topamax) | IonChBlocker | GABA_Ag |  |  |
|  | Valproic acid (Depakote) | IonChBlocker | GABA_Ag |  |  |
| Benzodiazepine | Alprazolam (Xanax/Niravam) | GABA_Ag |  |  |  |
|  | Chlordiazepoxide (Librium) | GABA_Ag |  |  |  |
|  | Clonazepam (Klonopin) | GABA_Ag |  |  |  |
|  | Diazepam (Valium) | GABA_Ag |  |  |  |
|  | Lorazepam (Ativan) | GABA_Ag |  |  |  |
|  | Temazepam (Restoril) | GABA_Ag |  |  |  |
|  | Triazolam | GABA_Ag |  |  |  |
|  | Quazepam (Doral) | GABA_Ag |  |  |  |
| Quasi-Benzodiazepine | Eszopiclone | GABA_Ag |  |  |  |
|  | Zolpidem | GABA_Ag |  |  |  |
| Psycho-Stimulant | Amphetamine / Dextroamphetamine (Adderall) | CA_Ag | MostlyNE |  |  |
|  | Armodafinil (Nuvigil) | CA_Ag | MostlyNE |  |  |
|  | Dexmethylphenidate (Focalin) | CA_Ag | MostlyNE |  |  |
|  | Dextroamphetamine (Dexedrine) | CA_Ag | MostlyNE |  |  |
|  | Lisdexamfetamine (Vyvanse) | CA_Ag | MostlyNE |  |  |
|  | Methylphenidate (Concerta, Ritalin, Methylin) | CA_Ag | MostlyNE |  |  |
|  | Modafinil (Provigil) | CA_Ag | MostlyNE |  |  |
| Lithium | Lithium |  |  |  |  |
| Other | Amlodipine | IonChBlocker |  |  |  |
|  | Baclofen | GABA_Ag |  |  |  |
|  | Buspirone | 5HT_Ag | Mostly5HT |  |  |
|  | Clonidine | CA_Ant |  |  |  |
|  | Ephedrine | CA_Ag |  |  |  |
|  | Prazosin | CA_Ant |  |  |  |
|  | Propranolol | CA_Ant |  |  |  |
|  | Tizanidine | CA_Ant |  |  |  |
|  | Verapamil | IonChBlocker |  |  |  |

*Abbreviations*: CA_Ag (Catecholaminergic agonist); CA_Ant (Catecholaminergic antagonist); 5HT_Ag (Serotoninergic agonists); Mostly5HT (Primarily serotonin action); 5HT&NE (Serotonin and norepinephrine action); MostlyNE (Primarily NE action); IonChBlocker (Voltage-gated ion channel blocker); GABA_Ag (GABA agonist).

**Supporting Table 2. Numbers and percentages of patients taking medications during acute rTMS treatment for depression, classified by MOA-based categories.**

| Medication Category | Number of Patients Taking Medication | Proportion (%) of the sample (n=181) |
| --- | --- | --- |
|  |  |  |
| CA_Ag | 126 | 69.6 |
| CA_Ant | 36 | 19.9 |
| 5HT_Ag | 118 | 65.2 |
| Mostly5HT | 95 | 52.5 |
| 5HTandNE | 38 | 21.0 |
| MostlyNE | 95 | 52.5 |
| IonChBlocker | 61 | 33.7 |
| GABA_Ag | 95 | 52.5 |

**Supporting Table 3. Results of linear regression examining MOA-based categories as predictors of week 2 outcome of rTMS for depression: Outcome was assessed as change in the Inventory of Depressive Symptomatology Self Report (IDS-SR30) (Rush et al., 1996) total score.**

**A. Models with baseline IDS-SR30 total score covariate only**

| Medication Category | Overall Model | Baseline IDS (p-value) | Medication Category (p-value) |
| --- | --- | --- | --- |
| CA_Ant | F=4.66; p=.011 | .147 | .012* |
| GABA_Ag | F=4.89; p=.009 | .045 | .010* |
| MostlyNE | F=3.58; p=.030 | .103 | .040* |
| CA_Ag | F=1.41; p=.247 | .095 | .902 |
| 5HT_Ag | F=1.40; p=.248 | .096 | .973 |
| Mostly5HT | F=1.43; p=.242 | .093 | .815 |
| 5HTandNE | F=1.40; p=.248 | .096 | .973 |
| IonChBlocker | F=1.51; p=.225 | .094 | .655 |

**B. Significant models with baseline anxiety and non-anxiety item total covariates, and additional covariates**

| Medication Category | Model Sig. | R^2^ | Covariates and significance | Medication Category Statistics | | | |
| --- | --- | --- | --- | --- | --- | --- | --- |
|  |  |  |  | Unstandardized β | Std. Error | t | p |
| GABA_Ag | .008 | .057 | Baseline non-anxiety IDS (p=.019) | 3.43 | 1.49 | 2.30 | .02* |
|  | .012 | .064 | Baseline non-anxiety IDS (p=.010), Baseline Anxiety (p=.265, N.S.) | 3.09 | 1.52 | 2.04 | .04* |
|  | .005 | .085 | Baseline non-anxiety IDS (p=.013), Baseline Anxiety (.513, N.S.), Age (p=.054, N.S.) | 3.19 | 1.51 | 2.12 | .04* |
| MostlyNE | .012 | .051 | Baseline non-anxiety IDS (p=.040) | -3.09 | 1.49 | -2.07 | .040* |
|  | .009 | .067 | Baseline non-anxiety IDS (p=.009), Baseline Anxiety (p=.097, N.S.) | -3.22 | 1.49 | -2.17 | .03* |
|  | .002 | .111 | Baseline non-anxiety IDS (p=.004), Total Meds (p=.040), Age (p=.023), Baseline Anxiety (p=.198, N.S.) | -4.44 | 1.64 | -2.71 | .007* |
| CA_Ant | .005 | .062 | Baseline non-anxiety IDS (p=.057, N.S.) | 4.77 | 1.90 | 2.51 | .01* |
|  | .005 | .074 | Baseline non-anxiety IDS (p=.018), Baseline Anxiety (p=.150, N.S.) | 4.64 | 1.90 | 2.45 | .02* |
|  | .004 | .090 | Baseline non-anxiety IDS (p=.021), Baseline Anxiety (p=.301, N.S.), Age (p=.093, N.S.) | 4.40 | 1.89 | 2.33 | .02* |
|  | .002 | .084 | Baseline non-anxiety IDS (p=.037), Age (p=.0501, N.S.) | 4.46 | 1.89 | 2.36 | .02* |

**Supporting** **Table 4. Medication effects in linear mixed model analyses examining MOA-based medication categories as predictors of change in symptom severity over time (weeks 2,4, and 6).**

| **MOA Category** | **Denominator df** | **F** | **p** |
| --- | --- | --- | --- |
| GABA_Ag | 178.188 | 4.144 | .043* |
| MostlyNE | 178.615 | 4.143 | .043* |
| CA_Ant | 177.390 | 3.343 | .069 |
| CA_Ag | 181.349 | .740 | .391 |
| 5HT_Ag | 179.152 | .000 | .990 |
| Mostly5HT | 178.008 | .216 | .643 |
| 5HTandNE | 178.008 | .216 | .643 |
| IonChBlocker | 177.254 | 1.382 | .241 |

All models included baseline severity as a covariate. Change in symptom severity was assessed using the Inventory of Depressive Symptomatology Self Report (IDS-SR30) (Rush et al., 1996).
